# Intracellular Dynamics of the Ubiquitin-Proteasome-System

**DOI:** 10.12688/f1000research.6835.2

**Published:** 2015-09-28

**Authors:** Maisha Chowdhury, Cordula Enenkel

**Affiliations:** 1 Department of Biochemistry, University of Toronto, Toronto, ON, M5S 1A8, Canada

**Keywords:** Proteasome, Storage Granules, Dynamics, Nuclear Transport, Ubiquitin System, Blm10, Importin, Karyopherin, Quiescence

## Abstract

The ubiquitin-proteasome system is the major degradation pathway for short-lived proteins in eukaryotic cells. Targets of the ubiquitin-proteasome-system are proteins regulating a broad range of cellular processes including cell cycle progression, gene expression, the quality control of proteostasis and the response to geno- and proteotoxic stress. Prior to degradation, the proteasomal substrate is marked with a poly-ubiquitin chain. The key protease of the ubiquitin system is the proteasome. In dividing cells, proteasomes exist as holo-enzymes composed of regulatory and core particles. The regulatory complex confers ubiquitin-recognition and ATP dependence on proteasomal protein degradation. The catalytic sites are located in the proteasome core particle. Proteasome holo-enzymes are predominantly nuclear suggesting a major requirement for proteasomal proteolysis in the nucleus. In cell cycle arrested mammalian or quiescent yeast cells, proteasomes deplete from the nucleus and accumulate in granules at the nuclear envelope (NE) / endoplasmic reticulum ( ER) membranes. In prolonged quiescence, proteasome granules drop off the nuclear envelopeNE / ER membranes and migrate as droplet-like entitiesstable organelles  throughout the cytoplasm, as thoroughly investigated in yeast. When quiescence yeast cells are allowed to resume growth, proteasome granules clear and proteasomes are rapidly imported into the nucleus.

Here, we summarize our knowledge about the enigmatic structure of proteasome storage granules and the trafficking of proteasomes and their substrates between the cyto- and nucleoplasm.

Most of our current knowledge is based on studies in yeast. Their translation to mammalian cells promises to provide keen insight into protein degradation in non-dividing cells, which comprise the majority of our body’s cells.

## Introduction

Proteolysis determines the half-life of proteins and thus controls protein homeostasis. If protein homeostasis is disrupted, the incidence of protein misfolding and neurodegenerative diseases such as Huntington’s, Parkinson’s and Alzheimer’s increases ( Ciechanover & Brundin, 2003).

In eukaryotic cells two highly conserved degradation pathways exist: under starvation long-lived proteins are preferentially degraded within the lysosome, an organelle with membranes which protect the surrounding cytoplasm against lysosomal hydrolases ( Fuertes *et al.*, 2003; Lee & Goldberg, 1996; Rendueles & Wolf, 1988); short-lived proteins are rather degraded by proteasomes, multimeric protease complexes which move between the nucleo- and cytoplasm ( Hershko & Ciechanover, 1998; Rock *et al.*, 1994). Proteasomal substrates are often nuclear proteins such as proteins regulating cell cycle progression (cyclin-dependant kinases and their inhibitors), gene expression (transcriptions factors), DNA damage and stress response; although, misfolded proteins occurring during protein synthesis in the cytoplasm are also rapidly degraded by the proteasome ( Kirschner, 1999; Vabulas & Hartl, 2005; von Mikecz, 2006). As a result proteasomal proteolysis serves to eliminate obsolete proteins which compete with functional proteins for binding partners and are prone to associate with irreversible and toxic protein aggregates ( Goldberg, 2003).

Here, we want to address the dynamics of proteasomes, which select their substrates by specific determinants such as poly-ubiquitylation, a covalently linked chain of ubiquitin molecules ( Finley, 2009). This ubiquitin-dependent proteolysis undertakes up to 90% of protein degradation in growing yeast and cultured mammalian cells and consumes considerable amounts of ATP, since the activation and conjugation of ubiquitin to the protein substrate as well as the unfolding and translocation of the protein substrate into the proteasome is ATP-dependent ( Coux *et al.*, 1996). Natively-disordered proteins also qualify as proteasome substrates and are cleaved without post-translational ubiquitin modification ( Erales & Coffino, 2014; Fishbain *et al.*, 2015; Liu *et al.*, 2003).

The advent of live cell imaging and GFP-labelling technologies in the 1990s ( Tsien, 1998) have greatly facilitated the study of proteasome dynamics in yeast and mammalian cells. Through these non-invasive techniques, the localization of the proteasome in growing yeast and highly proliferating cancer cells has been elucidated to be primarily nuclear ( Enenkel *et al.*, 1998; Laporte *et al.*, 2008; McDonald & Byers, 1997; Russell *et al.*, 1999). In line with this finding, increasing evidence in the literature suggests that certain misfolded proteins are imported from the cytoplasm into the nucleus solely for proteasomal degradation ( Park *et al.*, 2013; Prasad *et al.*, 2010). Conversely, transient nuclear proteins are exported into the cytoplasm for proteolysis, indicating a dynamic movement of proteasomal substrates between the nucleus and cytoplasm ( Chen & Madura, 2014a). Under nutrient deprivation and during transition from proliferation to quiescence, yeast proteasomes gather in proteasome storage granules (PSGs) at the nuclear envelope (NE)/endoplasmic reticulum (ER) membrane ( Enenkel, 2014; Knecht & Rivett, 2000; Wojcik & DeMartino, 2003). With prolonged quiescence PSGs seem to pinch off the NE/ER, but are not associated with specific organelles or any detectable membrane and are defined as motile spherical structures in the cytoplasm ( Laporte *et al.*, 2008). When cells resume growth, PSGs dissipate and proteasomes are rapidly imported into the nucleus to contribute their function in cell proliferation ( Laporte *et al.*, 2008). The mechanism of PSG formation and clearance is still unknown but seems to be conserved, since PSG-like structures are observed in primary cell lines of non-dividing neuronal cells and in immortalized cell lines of cancer cells, if they are chemically arrested in cell cycle progression ( Bingol & Schuman, 2006; Kaganovich *et al.*, 2008).

Our knowledge about proteasome dynamics in mammalian cells is poor. Thus, the focus of this review will be to critically integrate the literature about the dynamics of the proteasome, particularly based on studies in yeast. In our overview of the ubiquitin-proteasome system and common principles of nuclear transport, we cite and refer to original work and review articles written by investigators who did seminal work on these topics. In the paragraphs addressing detailed knowledge about proteasome dynamics we cite the original work.

## Discussion/analysis of the literature

### The Ubiquitin System

Ubiquitylation is a post-translational modification commonly associated with proteasomal protein degradation. At least four ubiquitin molecules are required for a poly-ubiquitin chain to be recognized by the proteasome ( Thrower *et al.*, 2000). Hershko and colleagues in the early 1980s showed that poly-ubiquitylation requires the ATP-dependent ubiquitin activation enzyme (E1), a family of ubiquitin conjugating enzymes (E2) and a family of ubiquitin protein ligases (E3) ( Hershko & Ciechanover, 1998). First, ATP hydrolysis is required to activate the AMP linkage to the C-terminal glycine of ubiquitin which enables the transfer of the ubiquitin moiety to the active site cysteine of the E1. Second, the E1-bound ubiquitin is linked to the active site cysteine residue of an E2 by transesterification. Finally, the E3 transfers the ubiquitin onto the substrate depending on the class of the E3 enzyme (RING, HECT and U-box ligases) ( Finley *et al.*, 2012; Harper & Schulman, 2006). Elongation of the ubiquitin chain is achieved as succeeding ubiquitin molecules form isopeptide linkages with specific lysines of the preceding ubiquitin ( Hershko & Ciechanover, 1998). Prior to degradation, deubiquitinating activities within the proteasome cleave and recycle the ubiquitin molecules from the substrates ( Crosas *et al.*, 2006; Hanna *et al.*, 2006; Lam *et al.*, 1997; Verma *et al.*, 2002). Deubiquitinating enzymes in the cyto- and nucleoplasm provide an additional level on the plasticity on the repertoire of proteasomal substrates ( Sahtoe & Sixma, 2015). Intriguingly, GFP-labelled ubiquitin and the E1, named Uba1, is primarily nuclear in growing yeast and mammalian cells suggesting that ubiquitin-dependent proteolysis mainly occurs in the nucleus ( Huh *et al.*, 2003; Salomons *et al.*, 2010; Sugaya *et al.*, 2014; Sugaya *et al.*, 2015).

### Proteasome assembly and composition

Composed of over 40 subunits, the proteasome is a protein complex of 2.5 MDa which consists of two main components: the 20S core particle (CP) and the 19S regulatory particle (RP) ( Coux *et al.*, 1996).

Proteasome configurations centered on the CP can have either one or two RPs but also one or two alternative proteasome activating complexes giving rise to a variety of proteasome complex configurations. Proteasome holo-enzymes engaged in the degradation of poly-ubiquitylated proteins require the RP, thus occur either as RP-CP or RP-CP-RP, also termed the 26S and the 30S proteasome, respectively ( Eytan *et al.*, 1989).

### Structure of the 20S Core Particle

The proteasome belongs to the family of threonine proteases and its maturation follows the concept of zymogen activation upon which proteases are activated, once they arrive at their destination. With a molecular mass of 700 kDa, the CP is composed of seven distinct α and β subunits, each of which form heptameric rings stacked into a barrel composed of two outer α rings and two inner β rings ( Groll *et al.*, 1997). The maturation of the CP involves the dimerization of two inactive precursor complexes, resembling two half-CPs. Half-CPs consist of an α ring and β ring with five of the seven β subunits synthesized with propeptides. With the dimerization of two half-CPs into the pre-holo CP, the autocatalytic processing of the propeptides is triggered and three β subunits contribute an active site threonine with different peptide cleavage specificities ( Li *et al.*, 2007; Ramos *et al.*, 1998). CP-dedicated chaperones, namely Pac/Pba/Poc 1-4 and Ump1, assist in CP assembly. Ump1 is a natively-disordered protein ( Kusmierczyk *et al.*, 2008; Ramos & Dohmen, 2008), which is buried inside the pre-holo CP and later on becoming the first substrate of the nascent CP ( Sa-Moura *et al.*, 2013; Uekusa *et al.*, 2014). The α rings are the key players in CP gating. Normally CP α rings are closed, unless they are opened by the RP to allow access of protein substrates into the proteolytic cavity ( Groll *et al.*, 2000).

### Structure of the 19S Regulatory Particle

As “gate keeper” of the CP, the RP is the best understood proteasome activator ( Rechsteiner & Hill, 2005). The RP is divided into two parts, the base and the lid subcomplexes. The RP base is composed of six ATPases of the triple A family (ATPases Associated with diverse cellular Activities), named Rpt1-6, and five non-ATPases, Rpn1, Rpn2, Rpn10, Rpn13 and Ubp6. The base Rpn subunits are involved in the recognition of the poly-ubiquitin chain and the Rpt ATPase subunits guide the unfolding and translocation of the polypeptide substrate into the CP ( Finley *et al.*, 1998). In contrast to the RP base subunits, the subunits comprising the RP lid are only of the non-ATPase class: Rpn3, Rpn5-9, Rpn11 and Rpn12 ( Glickman *et al.*, 1998). The main known function of the RP lid is the processing of poly-ubiquitin chains. Rpn11 contributes isopeptidase activity to recycle ubiquitin moieties from the protein substrates. Ubp6 also has ubiquitin hydrolase activity and assists in trimming poly-ubiquitin chains ( Crosas *et al.*, 2006; Hanna *et al.*, 2006; Lam *et al.*, 1997; Verma *et al.*, 2002). In principle, the RP ensures that only targeted substrates are degraded by the proteasome, thereby conferring the ubiquitin- and ATP-dependence towards proteasomal protein degradation.

Two competing models exist for RP assembly ( Funakoshi *et al.*, 2009; Le Tallec *et al.*, 2009; Park *et al.*, 2009; Roelofs *et al.*, 2009). The first posits that RP assembly occurs in modules independent of the CP with the help of four RP-dedicated chaperones, named Hsm3, Nas2, Nas6 and Rpn14 ( Funakoshi *et al.*, 2009). In contrast, the second model proposes that the CP serves as a scaffold for the heterohexameric ATPase ring of the RP base ( Park *et al.*, 2009). The second model, however, appears less likely with regard to X-ray structure analysis showing that the RP-dedicated chaperones hinder the association between the RP base and CP α ring ( Barrault *et al.*, 2012). The CP-independent assembly model is also supported by the finding that the assembly of RP base and lid can be reconstituted from recombinant proteins with the assistance of RP-dedicated chaperones but without the CP template ( Beckwith *et al.*, 2013). However, the CP could serve as a platform for RP base assembly, if RP-dedicated chaperones are limiting.

### Localization of the proteasome

At this point, it is important to acknowledge the importance of GFP labelling and the ease with which it has allowed localization studies to be conducted ( Enenkel, 2014; Groothuis & Reits, 2005). In our species of interest, *Saccharomyces cerevisiae*, which is an excellent model organism for eukaryotic cells, GFP labelling of proteasomes is achieved by homologous recombination techniques into the chromosomal locus to convert an endogenous proteasomal subunit to a GFP-tagged version ( Enenkel *et al.*, 1999; Laporte *et al.*, 2008; McDonald & Byers, 1997). Nearly all proteasomal genes are essential and could be modified by GFP fusions without interfering with their function; we prefer the CP subunits α4 and β5, the CP-dedicated chaperone Ump1, and the RP subunits Rpn1, Rpt1 and Rpn11 as GFP-labelled reporters, because their GFP fusion proteins are fully incorporated into the proteasomal subcomplexes. So far, ~30 subunits of the yeast proteasome were labelled with GFP. All of them reveal the same subcellular localization as thoroughly investigated by direct fluorescence microscopy in living yeast ( Laporte *et al.*, 2008). The localization studies based on GFP labelling agree well with previous studies using indirect immunofluorescence microscopy of endogenous proteasomes in fixed yeast cells ( Enenkel *et al.*, 1998; McDonald & Byers, 1997; Russell *et al.*, 1999; Wilkinson *et al.*, 1998), whereas direct and indirect localisations of proteasomes in higher eukaryotes are less consistent.

Seminal studies on proteasome localization in vertebrate cells were performed by Werner Franke’s and Wolfgang Baumeister’s laboratories in the early 1990s. Proteasomes were mainly detected in the nuclei of *Xenopus laevis* oocytes and cultured mammalian cells ( Amsterdam *et al.*, 1993; Hugle *et al.*, 1983; Kleinschmidt *et al.*, 1983). Later investigations reported a shift towards cytoplasmic proteasomes dependent on the type of the cell line and the density of the cell culture ( Wojcik & DeMartino, 2003). Proteasome localization also varies with the growth phase in yeast ( Laporte *et al.*, 2008; Weberruss *et al.*, 2013). In growing yeast at logarithmic phase (OD~1), proteasomes are primarily nuclear. During the transition from proliferation to quiescence and the entrance into stationary phase (OD>3), proteasomes deplete from the nucleus and accumulate at the NE/ER in membraneless droplet-like structures. These enigmatic structures of proteasome accumulations were initially observed by Isabelle Sagot and her co-workers, who coined the term proteasome storage granules (PSGs) ( Laporte *et al.*, 2008). With prolonged quiescence, one to two PSGs with a diameter of ~ 0.2 to 0.5 µm seem to pinch off the NE into the cytoplasm. The PSGs are motile and stable in yeast cultures and are kept in quiescence for several weeks. If quiescent yeast cells are allowed to resume growth by replacing the glucose-depleted medium with glucose-rich medium, the PSG rapidly clears and the proteasomes are relocated into the nucleus within a few minutes.

Studies with mammalian cancer cell lines also exploited GFP-labelling techniques and fluorescence recovery after photobleaching experiments. The experiments suggested that nuclear transport of GFP-labelled CP across the NE was inefficient. Only the mitotic breakdown of the NE and its reassembly after mitosis allowed nuclear uptake of proteasomes ( Reits *et al.*, 1997). However, this nuclear uptake mechanism cannot explain the predominant nuclear localization of proteasomes in yeast cells which divide without mitotic breakdown of the NE. Proteasomes are the second most abundant protein complexes in eukaryotic cells and require continuous synthesis within the cytoplasm and nuclear import during cell division ( Weberruss *et al.*, 2013). The most common route for protein complexes to cross the NE in an organism with closed mitosis is through nuclear pore complexes (NPC). Before we address this pathway for yeast proteasomes, we will shortly summarize the concept of nuclear transport through the NPC, a pathway conserved from yeast to human.

### Nuclear import in proliferating yeast cells

The NE is embellished with NPCs which regulate the entry of molecules into and out of the nucleus. Their principal function is to allow free diffusion of small molecules, such as water/ions/peptides, and to block non-specific translocation of macromolecules that exceed 40kDa or a diameter larger than 5nm ( Aitchison & Rout, 2012; Wente & Rout, 2010). Translocation of larger macromolecules requires specific interactions with the NPC. Protein cargoes therefore associate with soluble transport factors, called karyopherins/importins/exportins, that themselves interact with phenylalanine-glycine rich nucleoporins (FG-Nups) decorating the NPC ( Wozniak *et al.*, 1998). Importins and exportins identify their protein cargoes by nuclear localization sequences (NLSs) and nuclear export signals (NESs), which ensure their nuclear import and export, respectively. In the literature, there are variations of nuclear import and export signals, only some of which comply with the classical import/export concept. The classical concept applies for nuclear import of proteasomes. Thus, we will focus on the key components required for the classical pathway ( Gorlich & Kutay, 1999).

The classical nuclear import cycle starts with the association of the importin/karyopherin αβ heretodimer, called Srp1/Kap95 in yeast, with the cargo NLS. Two types of classical NLSs exist: the monopartite NLS which contains five basic amino acid residues and the bipartite NLS in which two clusters of basic residues are spaced by 10–12 indifferent residues. Importin α has the NLS-binding grooves, and importin β mediates the interaction with FG-Nups. The directionality of nuclear transport is dictated by the Ran-GTP/GDP gradient across the NE. Ran is a small GTPase, named Gsp1 in yeast. Ran exists in its GTP-bound state in the nucleus and in its GDP-bound state in the cytoplasm due to the actions of the Ran guanine nucleotide exchange factor (RanGEF) and the RanGTPase activating protein (RanGAP) in the nucleo- and cytoplasm, respectively ( Gorlich & Kutay, 1999; Moore & Blobel, 1993). In the nucleus, the cargo-importin αβ complex encounters RanGTP, which results in the release of the cargo ( Rexach & Blobel, 1995). Cargo-free importin αβ is recycled into the cytoplasm for the next round of nuclear import.

### Nuclear import of proteasomes during cell proliferation

**Figure 1.**
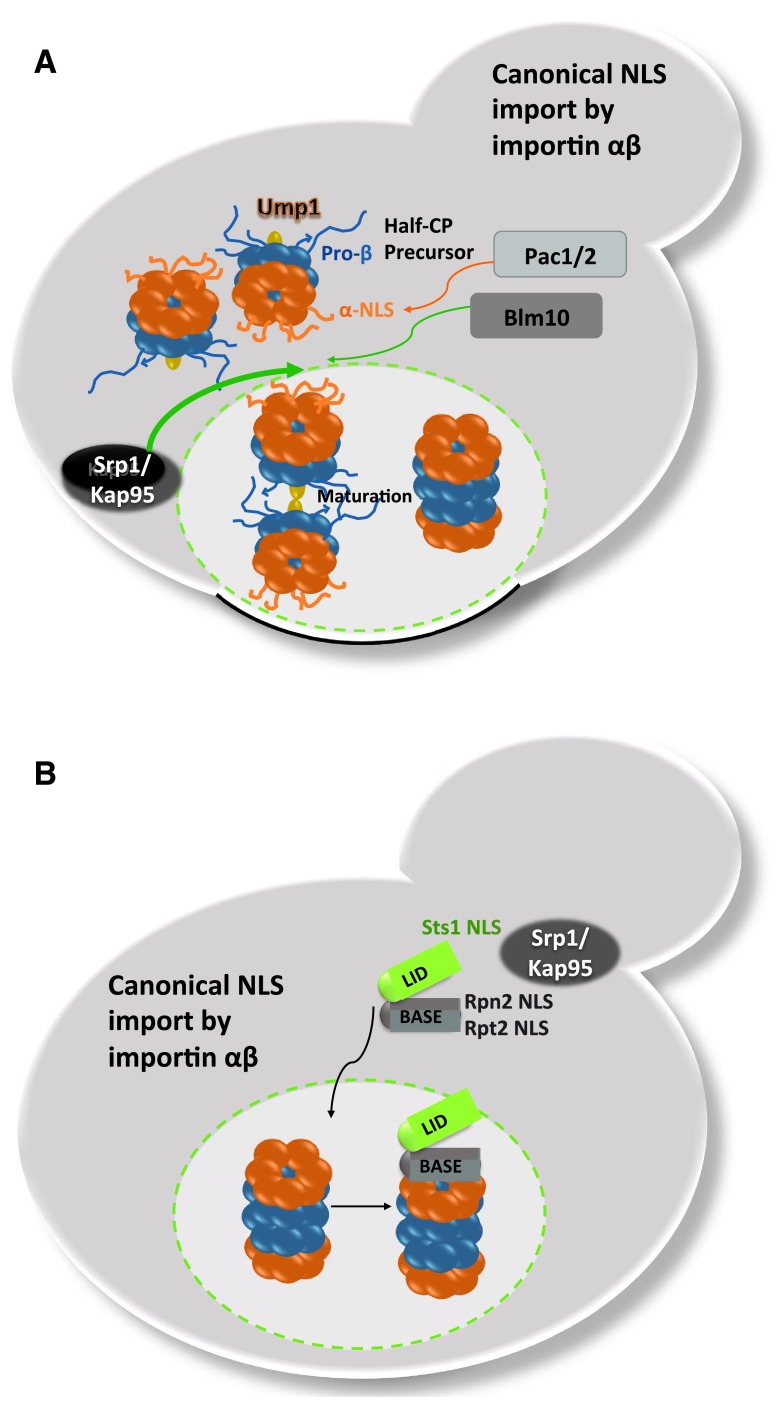
Model of nuclear proteasome assembly based on nuclear import of CP precursor complexes and RP subcomplexes in proliferating yeast cells. ( **A**) Ump1-containing CP precursor complexes are mainly imported into the nucleus by Srp1/Kap95, the classical importin/karyopherin αβ pathway. The α rings with the classical NLS are depicted in red. The β rings with propeptides are depicted in blue. The CP-dedicated chaperone and maturation factor Ump1 is depicted in yellow. The completion of CP maturation occurs in the nucleus with the degradation of Ump1. CP-dedicated chaperones Pac 1/2 are masking the NLS within the α ring, possibly preventing premature nuclear import. Blm10 serves as an alternative import receptor. ( **B**) Nuclear import of RP base and lid subcomplexes by the classical importin/karyopherin αβ pathway. Rpn2/Rpt2 and Sts1 confer classical NLS to the RP base and lid complex, respectively. Sts1 is short-lived and most likely degraded with nuclear RP-CP assembly.

Our studies in yeast strongly suggest that newly synthesized proteasomes are imported from the cytosol into the nucleus as inactive precursor complexes and that the maturation of nuclear CP proceeds to completion post-import ( Lehmann *et al.*, 2002). Although electron microscopy studies have shown that the NPC could expand to accommodate the longitudinal passage of the 30S proteasome, the permeability barriers towards macromolecules such as CP precursor complexes and RP assembly modules must be overcome by specific importins/karyopherins ( Pante & Kann, 2002). Several classical NLSs exist within the N-termini of distinct α subunits which were proposed to be either accessible rendering the CP in an import-competent conformation, or to be masked rendering the CP in an import-incompatible conformation ( Tanaka *et al.*, 1990). Indeed, recent EM structure analysis revealed flexible and less structured α ring surfaces in Ump1-associated CP precursor complexes ( Kock *et al.*, 2015; Wani *et al.*, 2015), consistent with our finding that importin α recognizes CP precursor complexes but not mature CP with closed α rings ( Lehmann *et al.*, 2002). Our model upon which CP precursor complexes are imported into the nucleus was supported by the following observations ( Figure 1A). First, when tagged with GFP, Ump1 is predominantly nuclear in spite of the fact that CP precursor complexes are assembled from nascent subunits in the cytoplasm. Second, in importin α mutants namely *srp1-49* but not in *srp1-31*, several groups found that the CP is mislocalized to the cytoplasm, providing another piece of evidence for the classical import pathway of proteasomes ( Chen & Madura, 2014b; Chen *et al.*, 2011; Lehmann *et al.*, 2002; Pack *et al.*, 2014; Wendler *et al.*, 2004). Unprocessed and incompletely processed β5 subunits, crucial determinants of CP precursor complexes and pre-holo-CP, respectively, accumulate in *srp1-49* mutants, while precursors of β5 subunits are hardly detectable in wild type cells ( Lehmann *et al.*, 2002). Third, when CP maturation is delayed by *UMP1* deletion, CP reporter proteins accumulate in the nucleus. Half of the reporter proteins is incorporated into incompletely matured CP, most likely the pre-holo-CP ( Fehlker *et al.*, 2003; Lehmann *et al.*, 2008). If mature CP were imported into the nucleus, CP precursor complexes would have accumulated in the cytoplasm of *ump1Δ* cells.

However, the CP-dedicated chaperones Pac/Pba/Poc 1-4 binding to the α ring are cytosolic ( Huh *et al.*, 2003). Particularly, Pac/Pba/Poc 1/2 seem to prevent premature nuclear import of CP precursor complexes by blocking the access to the NLSs within α subunits ( Kock *et al.*, 2015; Stadtmueller *et al.*, 2012), possibly allowing cytosolic CP maturation. Again, the deletion of Ump1 results in a predominant nuclear localization of Pac/Pba/Poc 1-4 supporting the model of nuclear import of CP precursor complexes (unpublished results, ( Le Tallec *et al.*, 2007)). Here, it is interesting to mention recent localization studies monitoring GFP-labelled β7 subunits in mammalian HeLa cells. This reporter subunit of the CP was found to be exclusively cytoplasmic but became nuclear upon DNA damage ( Kulichkova *et al.*, 2015). Possibly, the deletion of *UMP1* in yeast is comparable with DNA damage in human cancer cells and requests an abundance of nuclear proteasomes.

In the case of the RP, functional NLSs were identified in RP base subunits Rpn2 and Rpt2 and are recognized by importin α ( Figure 1B). The deletion of the Rpn2 NLS caused a temperature sensitive phenotype and mislocalizations of the RP base into cytosolic foci, whereas the deletion of the Rpt2 NLS was compensated by the presence of the Rpn2 NLS. At permissive temperatures, neither the Rpn2 nor the Rpt2 NLS deletion had severe impact on nuclear proteasome localization suggesting a redundancy of proteasomal NLSs ( Wendler *et al.*, 2004). Isono *et al.* (2007) later confirmed that Rpn2 provides a crucial NLS to aid nuclear import of the RP base and that the lid is separately imported. The nuclear import of the RP lid also requires importin α, though no classical NLS has been identified within RP lid subunits; rather Sts1, a short-lived protein that itself contains a classical NLS, associates with Rpn11 to facilitate nuclear import of the RP lid by importin αβ ( Chen *et al.*, 2011). In accordance, deletion of the Sts1 NLS has downstream effects on the nuclear localization of RP lid in addition to RP base and CP, which suggests that proteasomes could also be transported as holo-enzymes ( Chen & Madura, 2014b). In order to ensure comparable stoichiometry of proteasomal subcomplexes in the nucleus and similar kinetics by which they are imported into the nucleus, it is reasonable that importin αβ is used as common nuclear import receptor.

Recent fluorescence correlation spectroscopy studies also support the conclusion that proteasomes can be imported into the nucleus as holo-enzymes ( Pack *et al.*, 2014). However, the maturation state of the GFP-labelled proteasomes was unclear. Possibly, pre-holo-CP are the real nuclear transport intermediates which degrade Ump1 and Sts1 upon the arrival in the nucleus with the completion of proteasome maturation.

### Parallels between nuclear transport of proteasomes and ribosomes

Ribosome 40S and 60S subunits are the most abundant protein complexes in eukaryotic cells and are composed of more than 70 ribosomal subunits and four different ribosomal RNAs ( Marguerat *et al.*, 2012). Their assembly begins in the nucleolus and requires about 300 evolutionarily conserved nonribosomal trans-acting factors, which transiently associate with pre-ribosomal subunits at distinct assembly stages. Transport factors are required to import ribosomal proteins into the nucleus for pre-ribosomal subunit assembly and to passage pre-ribosomal subunits in a functionally inactive state through the NPC into the cytoplasm, where they undergo final maturation before initiating translation (for references see ( Gerhardy *et al.*, 2014)). Different GFP-tagged ribosomal protein and a pre-RNA reporter are established that reliably monitor the movement of pre-ribosomal particles from the nucleus into the cytoplasm in yeast ( Altvater *et al.*, 2014; Milkereit *et al.*, 2001; Tschochner & Hurt, 2003). Nuclear import of ribosomal proteins is mediated by importins belonging to the karyopherin β family ( Rout *et al.*, 1997). Export competent pre-ribosomal particles are separately exported by the general nuclear export factor Xpo1/Crm1. In addition, multiple trans-acting factors are engaged to shield the highly negative charge of the ribosomal RNA for entry into the disordered FG-Nups of the NPC. Most of the trans-acting factors are released and reused for another round of ribosome assembly. Failures in recycling a factor back into the nucleolus leads to its depletion resulting in delayed pre-ribosomal RNA processing, assembly defects and impaired nuclear export (for references on the original work see ( Gerhardy *et al.*, 2014)).

Certainly, proteasome and ribosome assembly differ as mature proteasomes do not contain RNA. However, parallels exist with regard to the tight coupling between assembly and transport of inactive precursor complexes.

### The Enigma of Proteasome Storage Granules

When cells experience nutrient exhaustion or enter quiescence, a drastic change in proteasome localization is observed. In prolonged quiescence, proteasomes deplete from the nucleus and reside in motile and reversible PSGs in the cytoplasm ( Laporte *et al.*, 2008). Upon addition of glucose, cells receive the signal to resume proliferation, and PSGs dissolve rapidly, and proteasomes are relocated in the nucleus. How PSGs are organized is not understood. Premature PSG formation in proliferating cells was found to depend on vacuolar ATPases and linked premature PSG formation with disregulation of the intracellular pH. In view of that, PSGs could serve as storage depots for mature proteasomes in quiescence, to protect the proteasome from cellular stress and elimination by autophagocytosis ( Peters *et al.*, 2013). The storage of proteasomes during quiescence would also alleviate energy-consuming synthesis of new proteasomes with cell proliferation ( Laporte *et al.*, 2008).

The formation of PSG-like structures is also observed by chemical inhibition of proteasomes in mammalian cells or temperature sensitive proteasome mutants in yeast, conditions which result in cell cycle arrest. In spite of the differences between chemically-induced cell cycle arrest and quiescence, inhibited proteasomes are sequestered into juxta nuclear quality control compartments (JUNQs), situated at the cytoplasmic side of the NE and behaving similar to PSGs. When the cell cycle-arrested mutants were allowed to resume growth at permissive temperatures or upon withdrawal of proteasome inhibition, JUNQs were seen to dissolve like the PSG. In the context of these studies poly-ubiquitylated proteins were found to be accumulated in the JUNQ. Thus, it was proposed that the JUNQ represents a major site for ubiquitin-dependent proteolysis ( Kaganovich *et al.*, 2008), though it has to be taken into account that JUNQ formation was induced by proteasome inhibition. The poly-ubiquitylated reporter proteins used in the studies on JUNQ functions by Kaganovich *et al.* (2008) were also detected within the PSG suggesting that JUNQ and PSG describe the same structure ( Weberruss *et al.*, 2013). All studies on the JUNQ and PSGs agree that these enigmatic structures serve protective functions. Their presence protects cells against proteo- and genotoxic stress and confers cell fitness during aging. Post-translation modifications such as N-acetylation also play a role in PSG organization, but their targets are unknown ( Saunier *et al.*, 2013; van Deventer *et al.*, 2015; Weberruss *et al.*, 2013).

### Nuclear import of proteasomes upon exit from quiescence

**Figure 2.**
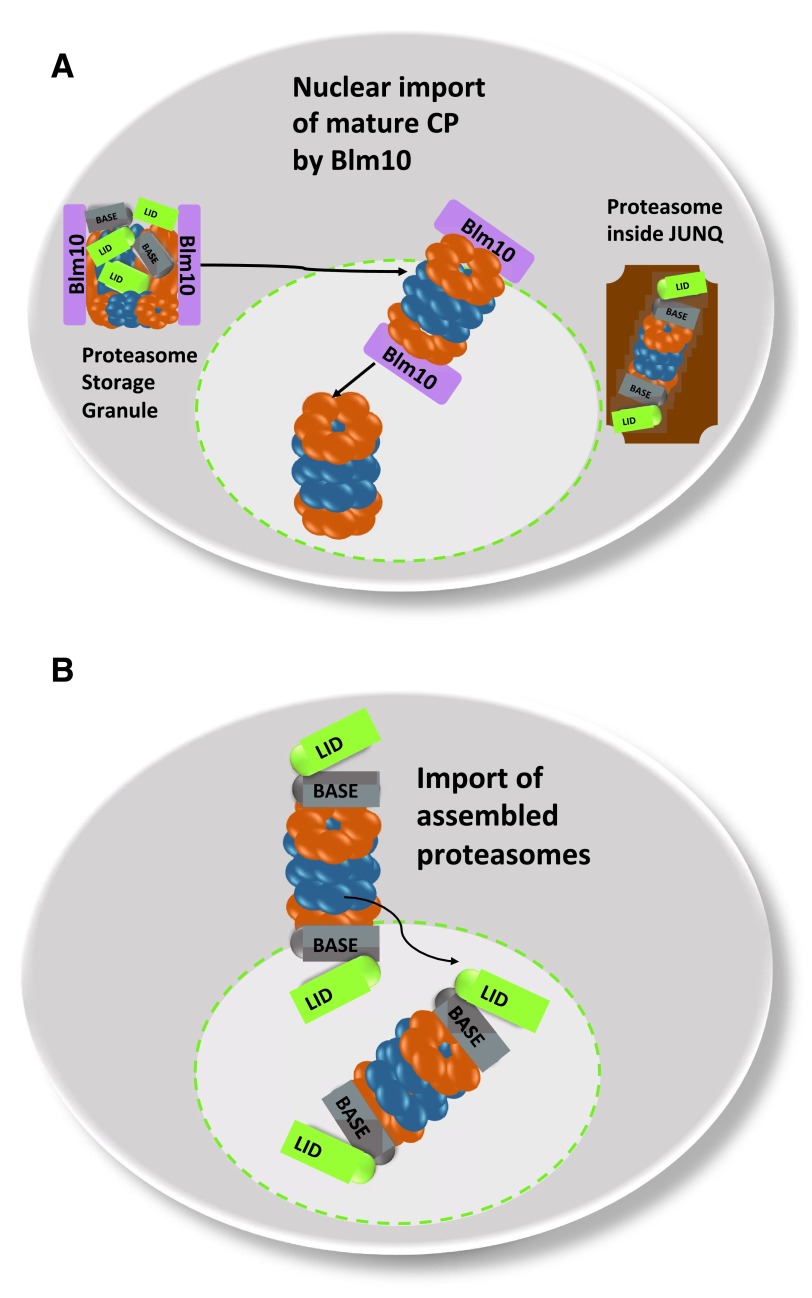
Model of nuclear import of mature proteasomes upon the exit from quiescence. ( **A**) In quiescence mature proteasomes are stored in PSG, reversible and motile granules in the cytoplasm. The PSG is formed at the NE/ER with the transition from proliferation to quiescence. The PSG clears with the resumption of growth and mature CP is imported into the nucleus by Blm10. In cell cycle arrested cells as induced by proteasome inhibition, proteasomes reside within JUNQ in the nuclear periphery. JUNQ rapidly clear with the release of proteasome inhibition. ( **B**) Assembled holo-proteasomes with RP-CP-RP configuration pass the nuclear pore.

Though the CP and RP co-localize in the PSG, they seem to be loosely associated. Conflicting reports exist about the stability of RP-CP assemblies in lysates of quiescent cells ( Bajorek *et al.*, 2003; Hanna *et al.*, 2012; Weberruss *et al.*, 2013). The finding that RP-CP assemblies are less stable coincides with the decline in ATP during quiescence as well as the reduced proclivity of the proteasome to degrade poly-ubiquitylated substrates. Instead of an association of the CP with the RP, most CP is seen interacting with Blm10, a conserved 240 kDa HEAT repeat protein ( Weberruss *et al.*, 2013). Upon exit from quiescence, the PSGs rapidly clear and mature proteasomes are imported into the nucleus within a few minutes. The imported proteasomes must be matured and assembled, as time does not permit the new synthesis of precursor complexes ( Laporte *et al.*, 2008). Here, Blm10 plays an important role and represents the first characterized nuclear transporter which particularly facilitates nuclear import of mature CP ( Figure 2A). Quiescent *blm10Δ* mutants exhibit a significant delay in resuming cell growth due to the deficit in mature CP in the nucleus. Furthermore, Blm10 binds FG-Nups and GTP-bound Ran and dissociates from the CP upon interaction with RanGTP, suggesting that Blm10 shares functional similarities with Kap95, the classical importin β ( Weberruss *et al.*, 2013). Along this line, Blm10 belongs to the HEAT repeat family with α-solenoid fold, a structural feature shared by β karyopherins/importins ( Huber & Groll, 2012). During cell proliferation, Blm10 is also expressed but to a much lesser extent ( Weberruss *et al.*, 2013). Only a minor fraction of the CP, pre-holo-CP and CP precursor complexes is associated with Blm10 in growing yeast. The Blm10-bound fraction significantly increases under geno-and proteotoxic stress suggesting a high demand for nuclear proteasomes under these growth conditions ( Doherty *et al.*, 2012; Fehlker *et al.*, 2003; Lehmann *et al.*, 2008). Since Blm10 associates with constitutively open or disordered CP α rings, Blm10 also plays a role in regulating α-ring gating during CP maturation ( Lehmann *et al.*, 2008). The wider α ring conformation of CP-precursor complexes seems to be preferentially bound to Blm10 and importin αβ by representing import intermediates. Thus, the Blm10-dependent import pathway complements the canonical nuclear import pathway, which also allows nuclear import of assembled proteasomes ( Chen *et al.*, 2011; Pack *et al.*, 2014), especially upon the exit from quiescence ( Figure 2B).

For the RP, the import pathway upon exit from quiescence is yet to be solidified. A possible candidate for a RP-dedicated nuclear import receptor is Rpn2 which exhibits a similar α-solenoid fold as Blm10 and importin β, all of which belong to the family of HEAT-repeat proteins ( Huber & Groll, 2012; Kajava, 2002).

## Conclusions

In this review, we discussed the recent literature on the dynamics of the ubiquitin-proteasome system with a major focus on the proteasome. During cell proliferation a high traffic volume of proteasomes and proteasomal substrates arises between the cyto- and nucleoplasm. In cell-cycle arrested and quiescent cells, proteasomes exit the nucleus and accumulate with poly-ubiquitylated proteins in motile and reversible PSGs in the nuclear periphery. While the basic concepts of nuclear import of proteasomes during cell proliferation and upon exit from quiescence are well explored, little is known about the nuclear export of proteasomes during the transition from proliferation to quiescence. We may wonder why proteasomes exit the nucleus during quiescence. Which kind of substrates will be available in the cytoplasm, once proteasomes are sequestered into the PSG? Possibly, PSG-resident proteasomes are starving for newly synthesized proteins which arise with the resumption of cell proliferation.

The dynamics of proteasomes and their substrates are fascinating and will inspire our discussions and experiments in the future.
