# Supplementary material for: Intracellular Dynamics of the Ubiquitin-Proteasome-System
Source: F1000Res. 2015 Sep 28;4:367. Originally published 2015 Jul 24. [Version 2] doi: 10.12688/f1000research.6835.2 (PMC4544378; doi:10.12688/f1000research.6835.2)
Supplement: Peer review history

# Peer review: Referee response for version 1

**DOI:** 10.5256/f1000research.7349.r9634

**Published:** 2015-08-20

**Authors:** Paula C. Ramos

**Affiliations:** 1 Institute for Genetics, University of Cologne, Cologne, Germany

The dynamics of proteasome composition and subcellular distribution is an important subject with far-reaching implication. Recently, substantial progress has been made on this subject, while many open questions remain. It is therefore of considerable value that Chowdhury and Enenkel have summarized and commented the current literature on this subject in this nice and timely review. I have only a few comments that should be easy to address:

1. In the second paragraph of the introduction it is stated that long-lived proteins are degraded in the lysosome, whereas short-lived proteins are degraded by the proteasome. While experimental data suggest that this is true for the bulk of the proteins in *S. cerevisiae* (e.g. Lee and Goldberg,1996), there is evidence from inhibitor studies with mammalian cells indicating that also long-lived proteins are among the proteasome substrates ( Rock *et al.* 1994). Thus, while the statement in the introduction goes in the right direction, it would be better to soften it a bit.
2. The sentence mentioning that Ump1 is a natively-disordered protein refers to papers from 2008. Reports that provided the first data supporting this notion, however, where published in 2013 by two independent groups (see below). Therefore, I would suggest to refer to these references in this context
  Ref.1: Sá-Moura B, S.A., Fraga J, Fernandes H, Abreu IA, Botelho HM, Gomes CH, Marques AJ, Dohmen RJ, Ramos PC, Macedo-Ribeiro S. Biochemical and Biophysical Characterization of Recombinant Yeast Proteasome Maturation Factor Ump1. Computational and Structural Biotechnology Journal 7, e201304006 (2013)
  Ref 2: Uekusa, Y. *et al*. Backbone ¹H, ¹³C and ¹⁵N assignments of yeast Ump1, an intrinsically disordered protein that functions as a proteasome assembly chaperone. Biomol NMR Assign (2013).
3. The structural data on 15S precursor complex were not obtained via cryo-EM but rather by negative stain EM (reference Kock et al. 2015).
4. When the authors discuss that the alpha ring of the 15S precursor complex is more relaxed and that would support the idea that the NLS would be more available for transportation from the cytoplasm into the nucleus, the authors have not considered or discussed the location of the chaperones Pba1-Pba2, that covers the surface corresponding to the pore of the 20S proteasome and thus would hide the NLS in this precursor form. I suggest to consider this point in the respective discussion and possibly to  add Pba1-Pba2 to the 15S precursor cartoon shown in figure 1.
5. It is mentioned (in the paragraph on “Nuclear import of proteasomes during proliferation”) that several groups found mislocalization of the CP to the cytoplasm, but no references are provided to support this notion.
6. It does not become entirely clear whether the authors propose that PSG and JUNQ are different names for one and the same compartment. Some of the remarks seem to suggest that, but this question is not explicitly raised and addressed. Maybe the authors could clarify this issue and their view on it a bit more.
7. While the authors describe PSGs as follows: “yeast proteasomes gather in proteasome storage granules (PSGs) at the nuclear envelope (NE) and endoplasmic reticulum (ER) membrane”, from which “with prolonged quiescence…  pinch off the NE into the cytoplasm”, in the study that originally coined the term PSG (Laporte et al. 2008), they were defined as “motile cytoplasmic structures” that are “not associated with specific organelles or any detectable membrane”. In fact, these PSGs were distinguished from locations of proteasomes at or close to the nuclear periphery (e.g. Figure 2B of that study). It thus appears as if Chowdhury and Enenkel use a different or wider definition of PSGs than the one used originally by Laporte et al., which is relevant to the previous issue (6.). If so, it would be helpful to propose and explain the reasons for such an expansion of the definition of PSG.
8. It is probably not ideal to describe PSGs as organelles.
9. While the entire process of ubiquitylation is ATP-dependent, of the three types of enzymes involved, only E1 is an ATP-dependent enzyme, E2 and E3 are not.

I have read this submission. I believe that I have an appropriate level of expertise to confirm that it is of an acceptable scientific standard.

# Response

**Published:** 2015-09-01

**Authors:** Cordula Enenkel

**Affiliations:** University of Toronto, Canada

Thanks for the helpful comments which were all considered in the revised version of our review.

With regard to point 7, Laporte *et al*. (2008) report the depletion of nuclear proteasomes during the transition from logarithmic to stationary phase and their accumulation at the NE / ER in dots close to the nuclear periphery. After 4-5 days in stationary phase the dots are mainly found in the cytoplasm and coined PSG (Figure 1 of that work).

I also clarified that PSG and JUNQ may be one and the same structure as shown by supplementary data in our work (Weberruss *et al. *2013).

# Peer review: Referee response for version 1

**DOI:** 10.5256/f1000research.7349.r9988

**Published:** 2015-08-14

**Authors:** Philip Coffino

**Affiliations:** 1 Department of Microbiology and Immunology, University of California, San Francisco, San Francisco, CA, USA

**Scope of this review and its intended readership**

The proteasome is a big and complex protein machine. This review centers on questions of its subcellular localization and where various steps in its assembly process happen. The basic question is inescapable and interesting: Proteins are made in the cytoplasm, but many or most proteasomes are found in the nucleus. How do large proteasome assemblies or subassemblies cross the nuclear membrane and where do they get put together (or sometimes taken apart)?

Presenting these matters requires background in two areas: 1. The structure and assembly of proteasomes and 2. Nuclear pore trafficking. Both are well-explored subjects, and some background on these matters needs to be presented. However, most readers of this review will bring some knowledge of these subjects, and wish to learn here about the specifics of the core subject, proteasome intracellular trafficking. I and perhaps other readers would prefer more on this and less (if space limitations require) of general background information. Additionally, how about comparing proteasome nuclear traffic and that of ribosomes? Ribosomes and proteasomes are the big machines found ubiquitously in eukaryotes. What common or distinct themes govern their subcellular movement and assembly?

**A Technical Question**

Much of the primary data supporting the conclusions described here depend on using various proteasome proteins fused to GFP in budding yeast. In interpreting these data it is critical to know whether or not such fusions alter kinetics or invoke alternate pathways compared to native controls. The paragraph headed **Localization of the proteasome** provides insufficient information related to this question.

**Minor considerations**

In the second paragraph of the Introduction it is said that “… long-lived proteins are degraded within the lysosome … short-lived proteins are degraded by proteasomes …” This seems too broad a claim- see Fuertes *et al. *(2003)[ref 1] for example- and should be modified or justified.

In the third paragraph of the Introduction: “Natively-disordered proteins also qualify as proteasome substrates and are cleaved without post-translational ubiquitin modification.”, the reference which follows is about something quite different and another more relevant reference should be used.

In the first paragraph under Discussion/analysis of the literature it is said that “At least four ubiquitin molecules … are required for a poly-ubiquitin chain to be recognized by the proteasome.” No longer regarded as so categorically true, but in any case reference should be made to Thrower *et al.* (2000) ^2^ for this specific claim.

Figure 3 conveys no information and can be dropped.

Page 3, first full paragraph, right column: Change “in compliance with our finding” to “consistent with our finding”.

In that same paragraph, perhaps the last sentence, “Third, when CP maturation is delayed by UMP1 deletion, all CP reporter proteins accumulate in the nucleus, although half of the CP is not fully matured and most likely exists as pre-holo-CP.” could be rewritten to clarify the intended meaning and how this supports the model of nuclear import of CP precursor complexes.

Page 5, left column, second full paragraph: “ …where proteasomes are relocated in the nucleus.” change to : “ …and proteasomes are relocated to the nucleus.”

Page 5 same paragraph: “ …and to be eliminated by autophagocytosis” change to “ …and elimination by autophagocytosis”.

I have read this submission. I believe that I have an appropriate level of expertise to confirm that it is of an acceptable scientific standard.

## References

[1] FuertesGVillarroyaAKnechtE: Role of proteasomes in the degradation of short-lived proteins in human fibroblasts under various growth conditions. *Int J Biochem Cell Biol.*.2003;35(5) : 10.1016/S1357-2725(02)00382-5 651-664 10.1016/S1357-2725(02)00382-5 Reference source 12672457

[2] ThrowerJSHoffmanLRechsteinerMPickartCM: Recognition of the polyubiquitin proteolytic signal. *Embo J*.2000;19(1) : 10.1093/emboj/19.1.94 94-102 10.1093/emboj/19.1.94 Reference source 10619848PMC1171781

# Response

**Published:** 2015-09-01

**Authors:** Cordula Enenkel

**Affiliations:** University of Toronto, Canada

Thanks for your constructive criticism of our review which addressed the intracellular dynamics of the ubiquitin-proteasome system. We reduced some background information, though nuclear transport of proteasomes will not be intelligible without profound knowledge of proteasome structure and assembly. We also omitted detailed informations about technical approaches, which were addressed in related reviews ( Burcoglu *et al.*, 2015). We answered your question with regard to direct and indirect localization studies using GFP technologies in living cells and antibodies in fixed cells. In yeast, the studies are highly consistent, while the data in mammalian cells are still difficult to reconcile.

We also appreciate your idea to compare nuclear transport and assembly of ribosomes and proteasomes. A chapter was added to address this point. However, we apologize that we can only refer to reviews about ribosome assembly and transport, because the citation of the original work would be beyond the scope of our review.

Your minor considerations were easily addressed.

# Peer review: Referee response for version 1

**DOI:** 10.5256/f1000research.7349.r9632

**Published:** 2015-07-31

**Authors:** Petra Wendler

**Affiliations:** 1 Gene Center Munich, Ludwig Maximilian University of Munich, Munich, Germany

This Review by Chowdhury and Enenkel nicely summarizes the current knowledge on the trafficking of proteasomes between cyto- and nucleoplasm. It's a very well written manuscript that gives a balanced and comprehensive view on localization studies on both, yeast and mammalian, proteasomes. I only have a few minor comments:

- The figures should be more informative. I would suggest to summarize all four figures in one or two figures, and to include/highlight all potential importers mentioned in the text (Kap95/Blm10/Rpn2). It would be tremendously helpful to add a figure summarizing the proteasomal localization data from yeast and mammalian cells. This figure should include which proteasomal subunits have been found where in the cell during which growth phase. It should also indicate the location of JUNQ and PSGs.
- Although the 20S chaperones Pba1/2 are mentioned, I am missing a discussion of their role in proteasomal localization. Binding of Pba1/2 blocs the access to the NLS of the alpha ring, as shown by Stadtmueller *et al. *(2012) and Kock *et al. *(2015). They thus seem to prevent nuclear import of proteasomal precursors.
- The authors incorrectly referred to "cryo-EM structure analysis of Ump1-associated CP precursor complexes" (Kock *et al*., 2015). This should be changed to "EM structure analysis…". Furthermore this point can be strengthened by also citing Wani *et al.* (2015).

I have read this submission. I believe that I have an appropriate level of expertise to confirm that it is of an acceptable scientific standard.

# Response

**Published:** 2015-09-01

**Authors:** Cordula Enenkel

**Affiliations:** University of Toronto, Canada

Thanks for your comments which were very supportive of our review.

According to your suggestions we renewed the figures and added more information about PSG and JUNQ. We also discussed possible functions of Pac1/2 in CP assembly and nuclear import.

Although it would be very helpful to summarize proteasomal localization data from yeast and mammalian cells in a table, it is difficult to reconcile the data in the mammalian system. Proteasome localizations depend on the mammalian cell line, the growth conditions, the fixation conditions and antibodies used in these studies. We prefer to mentioned these inconsistencies in the text. In yeast, proteasome localizations are independent of the reporter subunit as monitored by direct and indirect fluorescence microscopy.
